# Retraction Note: Cadherin 6 is activated by Epstein–Barr virus LMP1 to mediate EMT and metastasis as an interplay node of multiple pathways in nasopharyngeal carcinoma

**DOI:** 10.1038/s41389-022-00439-x

**Published:** 2022-10-17

**Authors:** L-L Zuo, J. Zhang, L- Z. Liu, Q. Zhou, S- J. Du, S- Y. Xin, Z- P. Ning, J. Yang, H- B. Yu, W- X. Yue, J. Wang, F- X. Zhu, G- Y. Li, J- H. Lu

**Affiliations:** 1 The Key Laboratory of Carcinogenesis of the Chinese Ministry of Health, Xiangya Hospital, Central South University, Changsha, 410080 China; 2 The Key Laboratory of Carcinogenesis and Cancer Invasion of the Chinese Ministry of Education, Cancer Research Institute, School of Basic Medical Science, Central South University, Changsha, 410078 China; 3 Hunan Key Laboratory of Nonresolving Inflammation and Cancer, The Third Xiangya Hospital, Central South University, Changsha, 410013 China; 4 Faculty of Chemical, Environmental and Biological Science and Technology, Dalian University of Technology, Dalian, 116024 China; 5 Department of Biological Science, Florida State University, Tallahassee, FL 32306 USA

Correction to: *Oncogenesis* 10.1038/s41389-017-0005-7, published online 22 December 2017

The Editor in Chief has retracted this article. After publication the authors reviewed this study and found errors which the authors then contacted the journal about in order to correct. However, due to the irregularities in figures 1A, 2G, 6C and E, figure 7 and supplementary figure 7B and 7D the Editor in Chief does not have confidence in the reliability of the results within this article.

L-L Zuo, J Zhang, L-Z Liu, Q Zhou, S-J Du, S-Y Xin, Z-P Ning, J Yang, W-X Yue, J Wang, F-X Zhu, G-Y Li and J-H Lu agree to this retraction. The editor was not able to obtain a current email address for H-B Yu.

